# Co-delivery of doxycycline and rifampicin using CdTe-labeled poly (lactic-co-glycolic) acid for treatment of *Brucella melitensis* infection

**DOI:** 10.1186/s13065-024-01200-8

**Published:** 2024-05-15

**Authors:** Saeideh Gohari, Seyed Mostafa Hosseini, Fatemeh Nouri, Rasoul Yousefimashouf, Mohammad Reza Arabestani, Mohammad Taheri

**Affiliations:** 1 Department of Microbiology, School of Medicine, Hamadan University of Medical Sciences, Hamadan, Iran; 2 Infectious Disease Research Center, Hamadan University of Medical Sciences, Hamadan, Iran; 3 Department of Pharmaceutical Biotechnology, School of Pharmacy, Hamadan University of Medical Sciences, Hamadan, Iran

**Keywords:** *Brucella melitensis*, PLGA, Doxycycline, Rifampicin

## Abstract

Brucellosis poses a significant challenge in the medical field as a systemic infection with a propensity for relapse. This study presented a novel approach to brucellosis treatment, enhancing the efficacy of doxycycline and rifampicin through the use of poly (lactic-co-glycolic) acid coupled with cadmium-telluride quantum dots (Dox-Rif-PLGA@CdTe). The double emulsion solvent evaporation method was employed to prepare Dox-Rif-PLGA@CdTe. The study scrutinized the physicochemical attributes of these nanoparticles. The impact of antibiotic-loaded nanoparticles on *Brucella melitensis* was evaluated through well diffusion, minimum inhibitory concentration (MIC), and cell culture. The chemical analysis results demonstrated a possibility of chemical reactions occurring among the constituents of nanoparticles. Assessments using the well diffusion and MIC methods indicated that the impact of free drugs and nanoparticles on bacteria was equivalent. However, the drug-loaded nanoparticles significantly decreased the colony-forming units (CFUs) within the cell lines compared to free drugs. In conclusion, the synthesis of nanoparticles adhered to environmentally friendly practices and demonstrated safety. The sustained drug release over 100 h facilitated drug accumulation at the bacterial site, resulting in a heightened therapeutic effect on *B. melitensis* and improved outcomes in brucellosis treatment. The application of these synthesized nanodrugs exhibited promising therapeutic potential.

**Supplementary Information:**

The online version contains supplementary material available at 10.1186/s13065-024-01200-8.

## Introduction

Brucellosis is a zoonotic infection caused mainly by the bacterium genus Brucella. This bacterial infection is spread to people via various methods, including consumption of contaminated food products, direct contact with sick animals, and aerosol inhalation. Humans are nevertheless susceptible to brucellosis, although they are unintended hosts. This ailment remains a significant worldwide public health issue and is the prevailing zoonotic infection. Human brucellosis continues to exert a considerable global impact, with the pathogen causing more than 500,000 infections each year. These intracellular infections can avoid detection by the host immune system and cause disease by multiplying within the host cells [1]. A chronic infection or disease relapse can be caused by bacteria residing in macrophages, which prevent cell death, adapt, grow, and evade the immune system [1, 2]. Therefore, managing brucellosis necessitates extended antibiotic treatment to minimize the risk of recurrence and the development of chronic disease [3, 4].

Brucella induces infection primarily through its ability to reside within macrophages, making it an intracellular pathogen. Brucella enters the host cell through a multi-step intracellular process that is facilitated by type four secretion systems of bacteria. The bacteria enter macrophages through endocytosis, establishing themselves within a Brucella-containing vacuole. This phase, known as early Brucella-containing vacuole (eBCV), occurs within 0–8 h post-infection due to the intrinsic endosomal nature of the bacterial vacuole [5, 6].

An effective treatment strategy requires a combination therapy due to the intracellular and slow-growth characteristics of *B. melitensis*, which incorporates at least one antimicrobial drug demonstrating robust penetration into intracellular spaces [3, 7]. In challenging situations, exploring a treatment strategy that combines doxycycline with aminoglycosides such as gentamicin or streptomycin is recommended. Alternatively, doxycycline and rifampicin are other effective approaches, such as adding supplementary gentamicin [3, 8, 9]. Additional options include trimethoprim, sulfamethoxazole, fluoroquinolones, and ciprofloxacin [8]. A doxycycline and streptomycin (SD) combination is currently considered the most effective therapeutic option for brucellosis, particularly in acute and localized manifestations. However, even though SD is often considered to be the most effective treatment, there is a 5–10% recurrence rate [10–12].

Given the constraints mentioned above, there is a pressing need to explore novel anti-brucellosis medications and enhance drug delivery methods for more effective brucellosis treatment [13–15].

The process of nano drug delivery involves the integration of nanoparticles with antimicrobial adjuncts in various forms, including dispersed, absorbed, or attached to the nanoparticle, which can modulate drug bioavailability and mitigate side effects [16].

Nanomaterials, spanning organic, inorganic, or hybrid compositions, exhibit dimensions smaller than a micron (< 900 nm) and are tailored to specific applications through varied synthesis methods. Nanoparticles, nanowires, and nanorods are some examples of these materials, and their structures encompass a broad spectrum, ranging from rod-like to fibrous or reticular networks, and encompass spheres characterized by either hollow or solid internal cavities with surfaces displaying smooth or irregular topographies. The remarkable properties of nanomaterials have catalyzed transformative advancements across numerous sectors, especially in medicine, where their distinctive attributes have spurred innovation and market evolution [17, 18].

Nanocarriers are becoming increasingly popular as drug delivery systems due to their enhanced stability during storage, improved precision in targeting diseased cells, sustained drug release, and superior drug encapsulation capacity [13, 19–21].

Nanocarriers offer the potential for enhanced treatments by reducing drug buildup within cells and diminishing the necessary dosage and dosing frequency. Studies have indicated that nanoparticles can effectively prevent drug degradation while regulating drug delivery and encapsulation [22–24].

PLGA is one of the most developed biodegradable polymers and its attractive attributes, including biodegradability and biocompatibility, have led to approvals by the FDA and the European Medicine Agency for drug delivery systems intended for parenteral administration. In addition, PLGA offers well-documented formulations and versatile production methods suitable for various drug types, whether hydrophilic or hydrophobic small molecules or macromolecules. Moreover, PLGA provides effective drug preservation and the potential for sustained release, making it a subject of significant interest [25–27].

PLGA has approval from the FDA and the EMA for its application in various human medication delivery systems. Nanoparticles possess several valuable attributes, including controlled and sustained drug release, minuscule sizes, and compatibility with cells and tissues. Furthermore, nanodrugs exhibit stability in the bloodstream and do not induce adverse effects like blood clotting, immune system activation, inflammation, or neutrophil activation. Additionally, these particles are biodegradable and effectively evade the reticuloendothelial system, making them an ideal choice for delivering a diverse array of pharmaceutical, protein, peptide, or nucleic acid compounds [28–30].

Therefore,, this research aimed to evaluate the effect of PLGA loaded with rifampicin and doxycycline on *Brucella millitensis* bacteria.

## Materials and methods

### Synthesis of CdTe-QDs

About 500 mg of sodium borohydride powder was dissolved in 7mL of water to make a solution. Then, 90 mg of Tellurium was added to the solution while argon gas was flowing, causing the solution to turn purple due to the strong interaction with oxygen. After two hours, the argon gas flow in the solution stopped as white sodium tetra borate powder precipitated. Thioglycolic acid (TGA) was added to a cadmium salt solution, followed by a dropwise addition of sodium hydrogen telluride (NaHTe) solution to create the NPs in the balloon. The initiation of nanoparticle formation commenced with the combination of Cd2 + and Te2- ions. Subsequently, the balloon was immersed in a water bath at 90 °C to promote the assembly of clusters [31].

### Synthesis of Dox-Rif-PLGA@CdTe

The double emulsion-solvent evaporation technique was employed to prepare Dox-Rif-PLGA@CdTe. Initially, 120 mg of PLGA polymer was dissolved in 15mL of chloroform, subjecting the mixture to stirring using a magnetic stirrer at 25 °C and 150 rpm for 3 h. Following this, 24 mg of doxycycline was added and dissolved in 5 ml of distilled water, and 24 mg of rifampicin in 5 ml of ethanol to the mixture, which led to the creation of the primary emulsion (W/O). The primary emulsion underwent further processing through homogenization with 2% PVA using ultrasonication equipment (specifically, Bandelin Sonopuls from Berlin, Germany). This homogenization took place for 1 min at 45% amplitude (20 W) with a controlled pulse rhythm (10 s on and 5 s off), resulting in the formation of the secondary emulsion (W/O/W). The emulsion, which included 200μL of CdTe-QD, was then meticulously added drop by drop into 20mL of cold water (at 4 °C) while magnetic stirring persisted for 30 min. Lastly, the Dox-Rif-PLGA@CdTe were isolated by a high-speed centrifuge, with the parameters set at 37,000 g for 20 min at 4 °C, followed by three washes with sterilized water [32].

### Characteristics of NPs

Dynamic Light Scattering was employed to assess the PS, PDI, and ZP of the Dox-Rif-PLGA@CdTe. These measurements were carried out using the Zetasizer Nano ZS 3600 apparatus [33].

### Morphology

The morphology of Dox-Rif-PLGA@CdTe was investigated using an electron microscope (FE-SEM). Then, 10 mg of the lyophilized Nano drug carrier was first mixed with 1 ml of water to initiate this analysis. Subsequently, 2μL of this suspension was deposited onto a glass surface. Following drying the suspension, a layer of gold was applied to the surface to prevent electrostatic charging during the examination process. Ultimately, the sample was examined using an FE-SEM (TSCAN in the Czech Republic) [34].

### Entrapment efficacy and drug loading

**Fig. 1:**
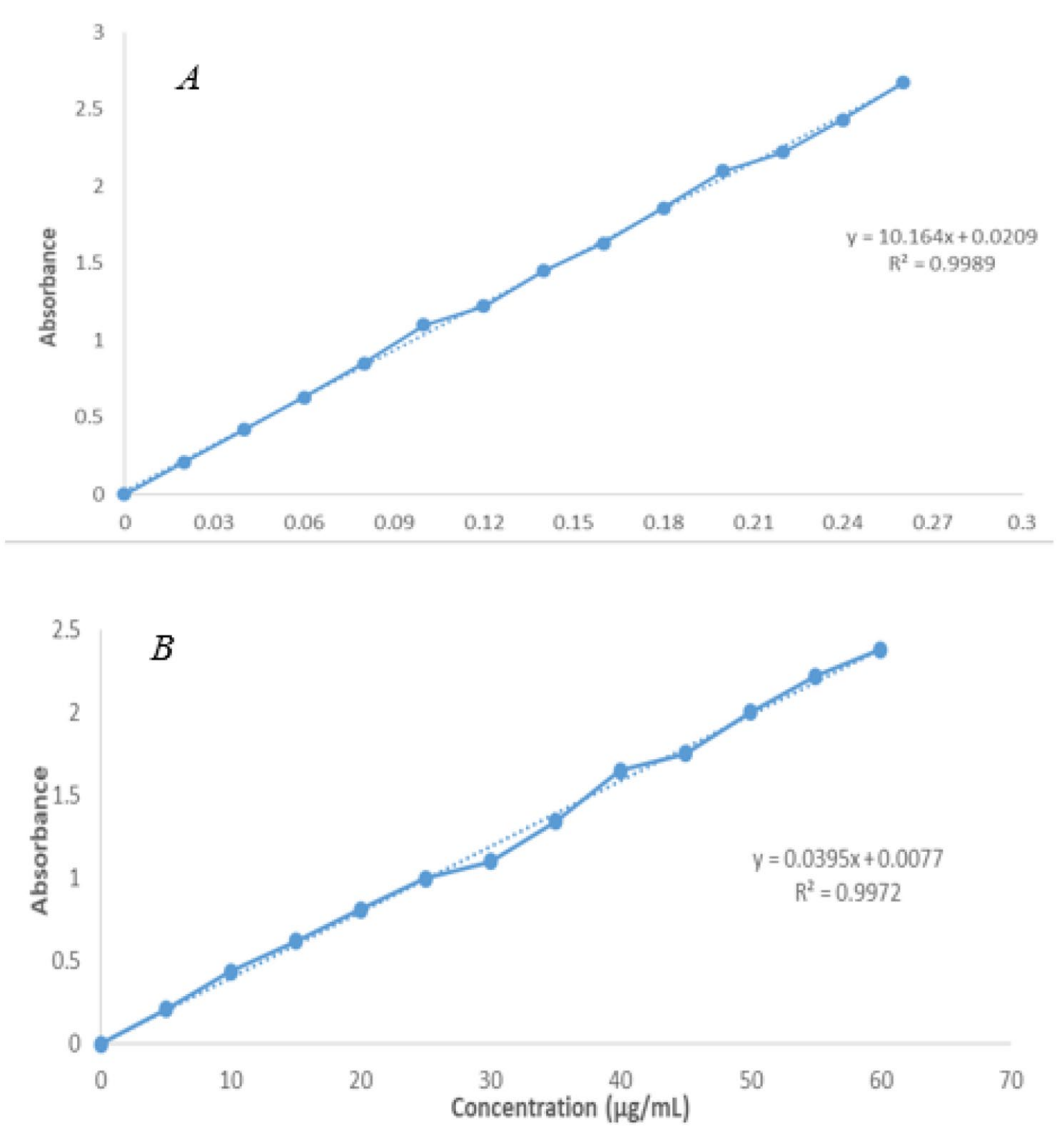
Standard curve with different antibiotic concentrations; (**A**) Doxycycline standard curve, (**B**) Rifampicin standard curve

An indirect method was utilized with a spectrophotometer to determine the concentrations of doxycycline and rifampicin present in Dox-Rif-PLGA@CdTe, following established guidelines from the literature. Initially, 5 mg of freeze-dried nanoparticles were mixed with 1 ml of distilled water and stirred using a vortex. Subsequently, the mixture was centrifuged at a speed of 37,000×g for 20 min at 4 °C. The resulting supernatant was then assessed at a wavelength of 283 and 331 nm, and the drug concentration was determined using a standard curve (Fig. 1) [34].

### Determination of stability of NPs

The stability of the NPs was systematically assessed at predefined intervals. The size, ZP, and PDI were monitored at six time points: 0, 2, 4, 6, 8, and 12 months after the lyophilization process. Additionally, the quantities of doxycycline and rifampicin loaded were measured within the NPs at these same time intervals using a spectrophotometer [35].

### Determination of drug release

The freeze-dried Dox-Rif-PLGA@CdTe were accurately weighed and placed inside a dialysis bag (molecular weight 12,000, specifically, Dialysis tubing from Sigma Chem. Co., Missouri, USA). This dialysis bag containing the nanoparticles was immersed in a 40 ml release solution consisting of a PBS buffer with a pH of 7.4. The setup was then subjected to magnetic stirring at 100 rpm and kept at 37 °C. About 1 ml of samples was collected from the release medium at predetermined intervals, and a spectrophotometer was used to determine the doxycycline and rifampicin content. A similar procedure was performed to compare the results obtained from free doxycycline and rifampicin, where free doxycycline and rifampicin were placed inside dialysis bags and immersed in the same release medium. Repeated samples were drawn from the medium at these same intervals for analysis. An equal volume of fresh medium was replenished after each sample was taken from the medium [36].

### FTIR and DSC analysis

The thermal properties and components of the optimal formulation were evaluated using differential scanning calorimetry (DSC). Thus, a freeze-dried powder of the ideal formulation, weighing between 5 and 10 mg, and its constituent elements, including free PLGA, doxycycline, rifampicin, and DOX-RIF-PLGA@CdTe, were subjected to testing under a nitrogen gas atmosphere (flowing at a rate of 80mL/min). This analysis was conducted over a temperature range from 20 to 400 °C, with a heating rate of 5 °C per minute [37].

The chemical structure of the ideal formula was obtained by separately mixing potassium bromide (KBr) with the lyophilized powder of the optimal formula and each constituent element. Subsequently, FTIR spectroscopy was performed in the mid-infrared (IR) spectrum from 4000 to 400 cm^− 1^ [37].

### Agar well diffusion and MIC

The antibacterial properties of Dox-Rif-PLGA@CdTe, Dox-PLGA, Rif-PLGA, and free drugs were assessed using the well diffusion method and Minimum Inhibitory Concentration (MIC) following CLSI guidelines. The initial step involves dissolving them in double-distilled water (DDW) and then subjecting the solution to sterilization through a 450 nm filter to prepare lyophilized nano-drug carriers appropriate for examining bacterial phenomena. In the well diffusion method, sterile swabs were utilized to culture *B. melitensis* on Mueller-Hinton agar. Then, wells were created in the agar medium, each with an 8 mm diameter, which was filled with 100mL of nano-drug carriers and free drugs at various concentrations in micrograms per milliliter. The plates were incubated for three days. The turbidity of bacterial growth was measured in each well to evaluate the antibacterial efficacy of the nanodrug carriers.

Due to the potential challenges of uniformly dispersing nano drug carriers in solid media, the results derived from the good diffusion technique were validated by the broth microdilution (BMD) method using 96-well polypropylene tissue culture plates. Here, 100 μl of Mueller Hinton’s broth culture medium and 100 nano-drug carriers and free drugs were added to each well, and serial dilutions were performed. Then, 10 μl of 5$\times$10^6^ colony-forming units (CFU/mL) of B. Melitensis were added to the wells and incubated at 37 °C for 24, 48, and 72 h. After incubation, the concentration at which no bacteria grew was determined and reported as the MIC [13].

### MTT assay

The MTT assay test was carried out to evaluate the cytotoxicity effect of Dox-Rif-PLGA@CdTe. The toxicity effect of Dox-Rif-PLGA@CdTe, free doxycycline, free rifampicin, and free PLGA was investigated using an MTT assay kit (kiazist, Iran) in Mouse monocyte-macrophage cells J774A.1 (ATCC TIB-67; BALB/c Mouse, hematopoietic, macrophage-like; Pasteur Institute, Tehran, Iran), and the test was performed based on the guidelines of the kit. The positive control (which was 100% alive) that was absorbed into the cells was used to calculate the vitality of the cells [13].

### In vitro infection assay

The study aimed to evaluate the effectiveness of Dox-PLGA, Rif-PLGA, and Dox-Rif-PLGA@CdTe in comparison to free doxycycline and rifampicin in killing intracellular *B. melitensis* within J774A.1 cells. Cells were cultured for 24 h before infection to conduct this assessment. When the cell confluence reached 90% in each well, the cell count was increased to 5,000 cells per well. Subsequently, 5 × 10^5^*B. melitensis* bacteria were added to each well, maintaining a 1:100 (cell/bacteria ratio), and incubated for 1 h to allow phagocytosis.

Cells had to be cleansed twice with PBS after 24 h of infection. Then, 100 μl of cell culture medium with FBS was introduced into the wells. Subsequently, various dilutions (12.5, 25, 50, 100, and 200 μl) of Dox-PLGA, Rif-PLGA, Dox-Rif-PLGA@CdTe, free doxycycline, free rifampicin, and free PLGA (blank) were added. These mixtures were then incubated for 24, 48, and 72 h at 37 °C in the presence of 5% CO2.

The cells were subjected to lysis and subsequent dilution. The resulting lysate was subsequently cultured using a bacterial culture medium. The number of Colony Forming Units (CFUs) was determined after an incubation period of three days at 37 °C [38].

## Results

### Nanoparticle’s characterization

In Dox-Rif-PLGA@CdTe (F4), the size, PDI, and ZP had been 239.9 ± 25 nm, 0.374 ± 0.015, and − 19.2 ± 1.2mV, respectively (Table 1).

**Table 1:** Technological and materials parameters of different formulation

	Formulation	doxycycline (mg)	rifampicin(mg)	PLGA (mg)	size	PDI	Zeta potential (mV)	Average encapsulation of drugs (%)	Average load of drugs (%)
Before Lyophilize	F1	6	6	30	554.6	0.321	-16.3	91.2	15.3
	F2	12	12	60	489.2	0.314	-14.3	93.6	13.8
	F3	12	12	90	354.8	0.478	-17.7	89.8	16.5
	**F4**	**24**	**24**	**120**	**239.9**	**0.374**	**-19.2**	**94.1**	**17.2**
	F5	24	24	90	376.7	0.343	-15.3	91.6	14.3
	F6	12	12	30	432.3	0.312	-16.4	86.8	14.8
	F7	24	24	60	523.1	0.390	-14.6	87.8	12.2
	F8	48	48	240	677.3	0.458	-13.2	90.4	10.3
After Lyophilize	F1	6	6	30	673.4	0.416	-15.9	90.6	15.1
	F2	12	12	60	524.3	0.386	-13.7	92.4	11.7
	F3	12	12	90	397.2	0.503	-16.5	84.7	11.3
	**F4**	**24**	**24**	**120**	**336.4**	**0.397**	**-17.6**	**85.3**	**16.2**
	F5	24	24	90	426.9	0.385	-14.2	90.0	12.4
	F6	12	12	30	516.2	0.425	-15.9	82.3	13.8
	F7	24	24	60	564.7	0.434	-13.3	81.6	11.7
	F8	48	48	240	694.2	0.553	-11.4	88.2	9.8

### Morphology

**Fig. 2:**
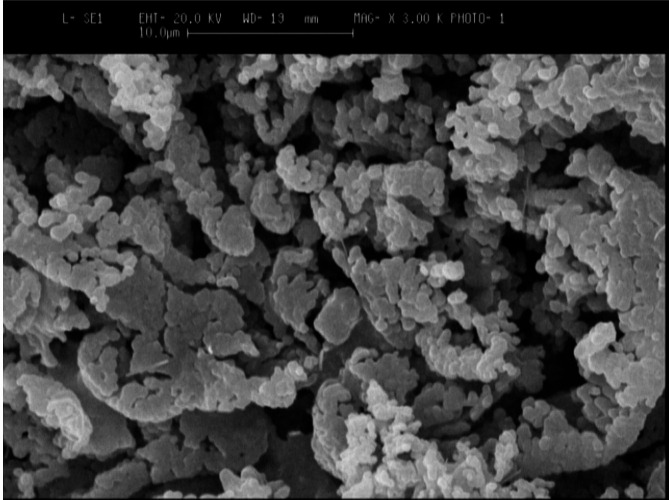
Field emission scanning electronic microscope images of Dox-Rif-PLGA@CdTe

The results of the Dox-Rif-PLGA@CdTe morphology investigation was shown using FE-SEM (Fig. 2). According to the image, the particles are spherical, uniformly dispersed, and of the same size.

### Drug loading and entrapment efficiency

The amount of rifampicin and doxycycline loaded in PLGA in formulations ranged from 10.3 to 17.2%, with entrapment varying from 86.8 to 94.1%. Loaded and encapsulated doxycycline and rifampicin levels for the optimal formulation of Dox-Rif-PLGA@CdTe (F4) were 17.2% 2.3 and 94.1% 2.4, respectively (Table 1).

### Stability of Dox-Rif-PLGA@CdTe

Nanoparticle particle size, PDI, and ZP were measured every two months to a year after production (Table 2). The data revealed that the size of the nanoparticles remained nearly constant until six months after manufacture, with only a minor fluctuation in size. These diameters expanded from 239.925 to 389.532 nm after a year. Changes in PDI and ZP were not statistically significant.

**Table 2:** Size, PDI, and ZP of Dox-Rif-PLGA@CdTe during the stability study (mean ± SD, *n* = 3)

Formulation	Technological parameters	Time (months)					
		0	2	4	6	8	10
Dox-Rif-PLGA@CdTe (F4)	Size (nm)	239.9 ± 25	261.3 ± 34	276.4 ± 23	299.8 ± 37	362.4 ± 39	389.5 ± 32
	PDI	0.374 ± 0.015	0.346 ± 0.013	0.321 ± 0.015	0.334 ± 0.012	0316.±0.016	0.291 ± 0.011
	ZP^*^ (mV)	-19.2 ± 1.2	-17.2 ± 2.3	-16.3 ± 2.1	-17.5 ± 2.9	-19.1 ± 4.1	-17 ± 3.6

### Drug release

**Fig. 3:**
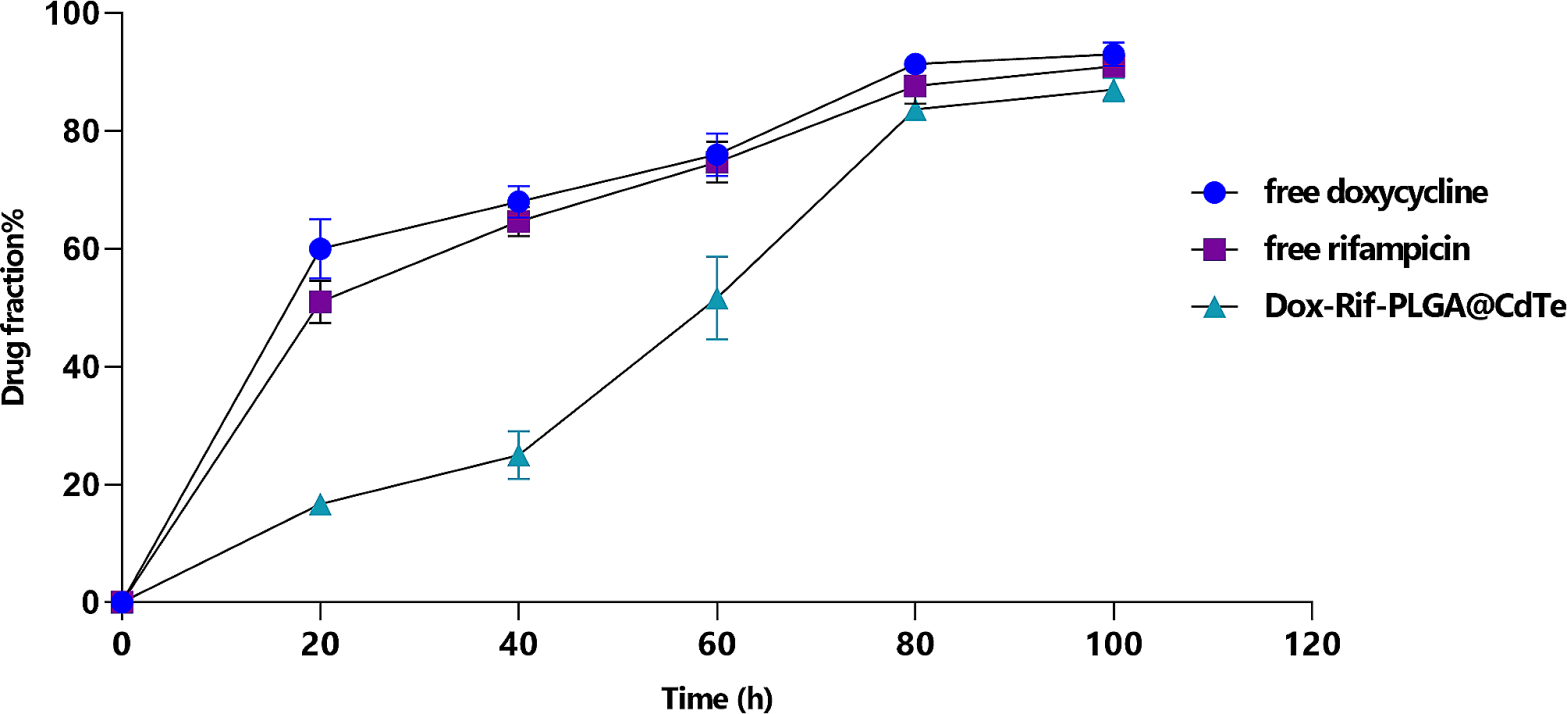
Release profiles of free doxycycline and free rifampicin from the Dox-Rif-PLGA@CdTe (No = 3)

A release test was conducted over 120 h in pH 7.4 PBS buffer for free doxycycline, free rifampicin, and Dox-Rif-PLGA@CdTe. In the initial 20 h, there was a significant swift release of free-doxycycline and free-rifampicin, accounting for approximately 60% of the drug. In contrast, during this period, only 18% of the drug was released from Dox-Rif-PLGA@CdTe. In total, 100 h were required for approximately 82% of the drug to be released from the Dox-Rif-PLGA@CdTe formulation, as illustrated in Fig. 3.

### FTIR analysis

**Fig. 4:**
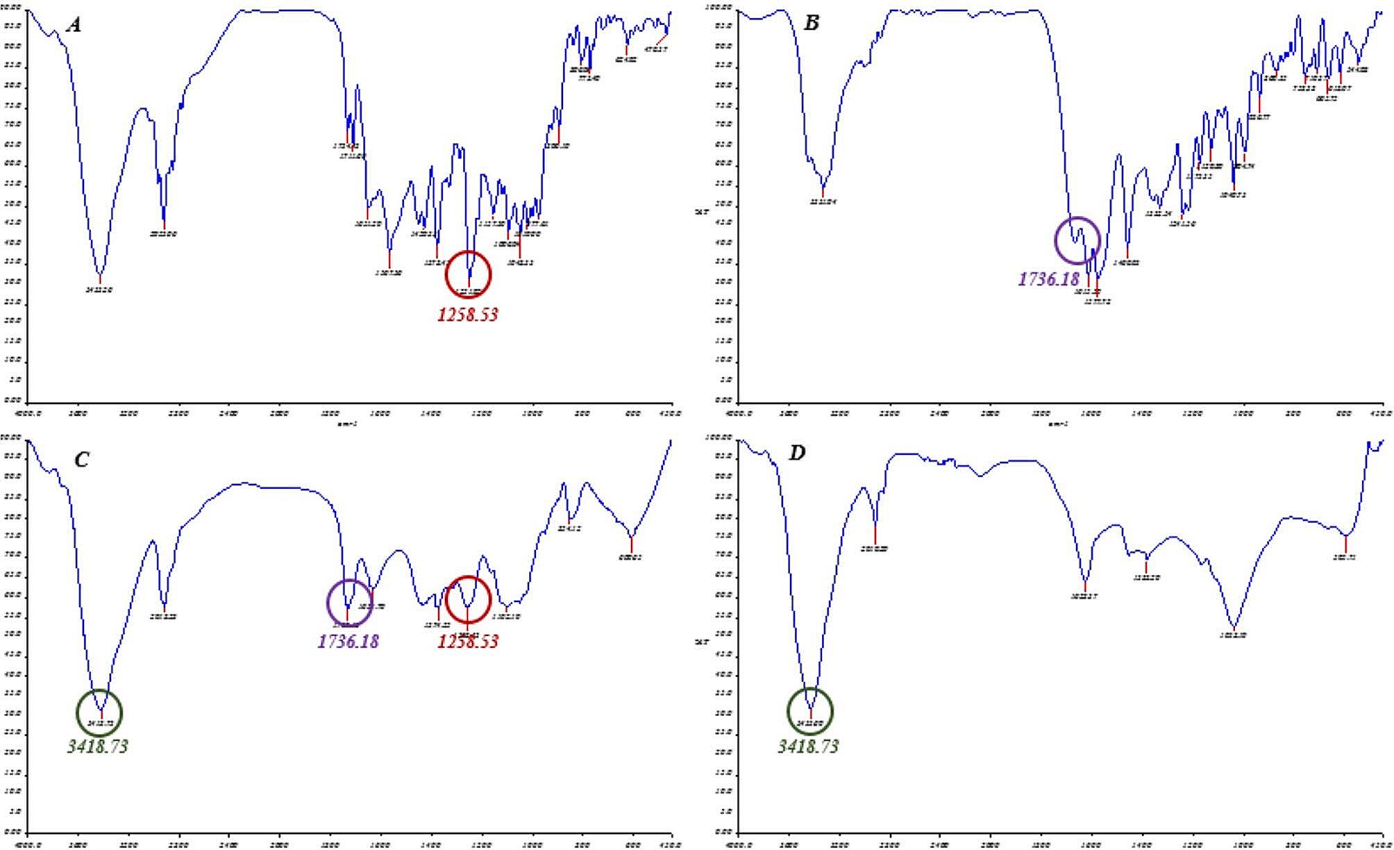
FTIR spectra of (**A**) rifampicin, (**B**) doxycycline, (**C**) Dox-Rif-PLGA@CdTe, and (D) free PLGA

Figure 4 exhibits the FTIR spectra of PLGA, Dox-Rif-PLGA@CdTe, doxycycline, and rifampicin. As shown in Fig. 3, adding rifampicin and doxycycline to PLGA nanoparticles shields the drug’s absorption peaks at 1258.53 and 1736.18 cm^− 1^. When comparing the spectra of PLGA and Dox-Rif-PLGA@CdTe, it is evident that the prominent PLGA absorption peaks at 3418.73 cm^− 1^ appeared in the Dox-Rif-PLGA@CdTe.

### DSC analysis

DSC (Differential Scanning Calorimetry) analyses were conducted on doxycycline, rifampicin, PLGA, and Dox-Rif-PLGA@CdTe to examine the recrystallization and melting characteristics of Dox-Rif-PLGA@CdTe, which are provided in Supplementary Fig. 1. In the DSC thermogram of pure PLGA, a melting process was observed at 65 °C. Interestingly, the melting points of the physical mixture and the Dox-Rif-PLGA@CdTe closely resembled pure PLGA. However, a slight alteration in the melting process of PLGA was noted when combined with the drug, consistent with previous research findings. Distinct sharp endothermic peaks were observed at 230 and 187 °C in the thermograms of doxycycline and rifampicin, respectively. These characteristics in other components indicated a minor melting point shift in the physical mixture and the Dox-Rif-PLGA@CdTe case. Remarkably, no changes were observed in the positions of the endothermic peaks for doxycycline, rifampicin, the physical mixture, and Dox-Rif-PLGA@CdTe. A Dox-Rif-PLGA@CdTe thermogram with no distinct melting peaks indicates that doxycycline and rifampicin molecules are effectively stabilized within the PLGA matrix, implying that no free crystals are present in the formulation.

### Agar well diffusion and minimum inhibitory concentration MIC

**Fig. 5:**
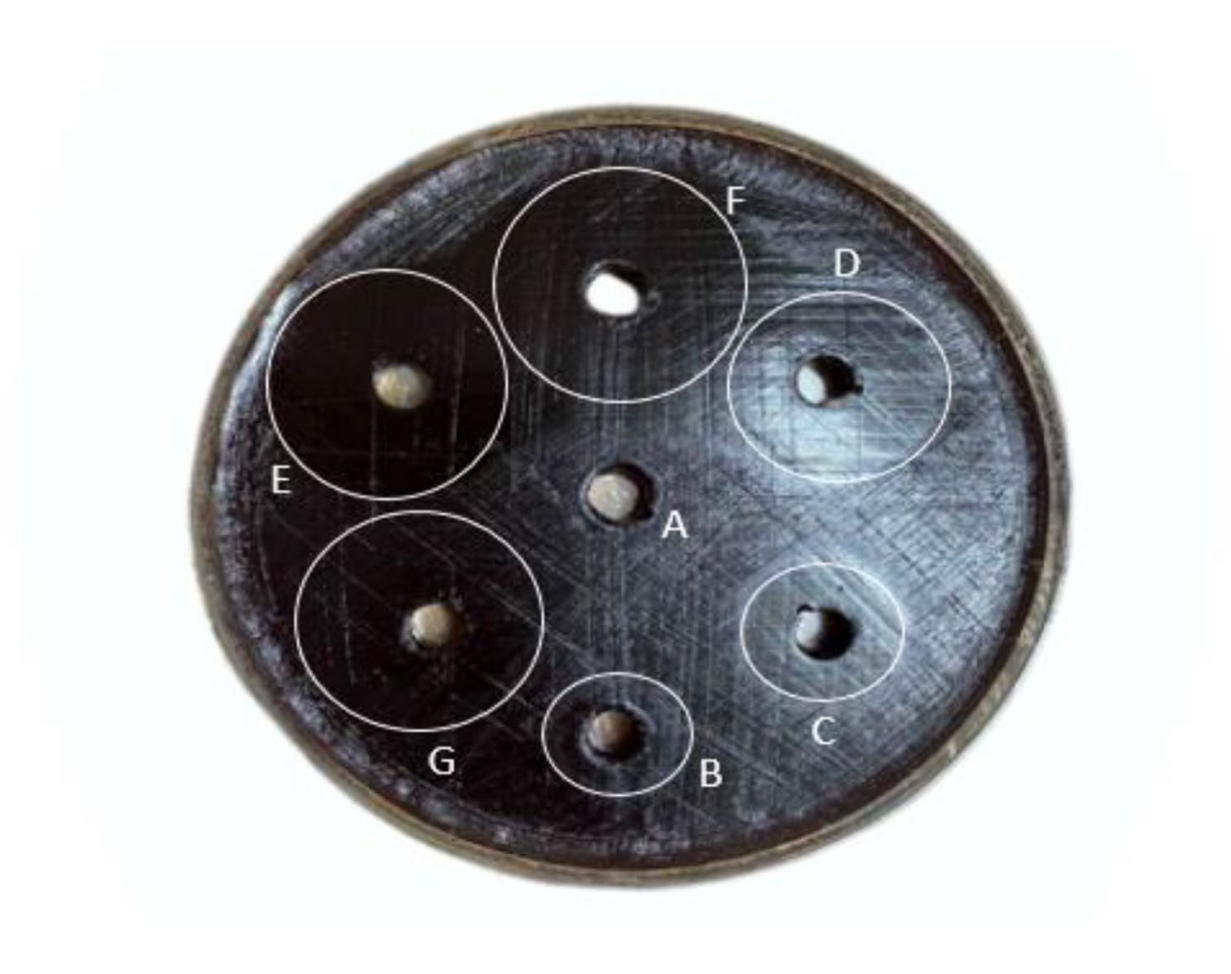
Different concentrations of Dox-Rif-PLGA@CdTe on Mueller Hinton Agar medium against *Brucella millitensis*; (**A**) Control (normal saline), (**B**) 3.125 μg/ml, (**C**) 6.25 μg/ml, (**D**) 12.5 μg/ml, (**E**) 25 μg/ml, (**F**) 50 μg/ml, (**G**) 100 μg/ml

Table 3; Fig. 5 show the well diffusion and MIC test, respectively. Free doxycycline and rifampicin performed better in both procedures at 24 and 48 h than Dox-Rif-PLGA@CdTe. However, the impact of free medicines on the MIC test diminished after 72 h of incubation. Furthermore, the medication was released from Dox-Rif-PLGA@CdTe after 72 h of incubation, gradually expanding the inhibitory zone of the compound.

**Table 3:** Results of the MIC test

MIC value (μg/ml)			
Time (h)	Dox-Rif-PLGA@CdTe	Free-Dox	Free-Rif
24 h	50	3.125	6.25
48 h	25	3,125	6,25
72 h	3.125	6,25	12,5

### MTT assay

**Fig. 6:**
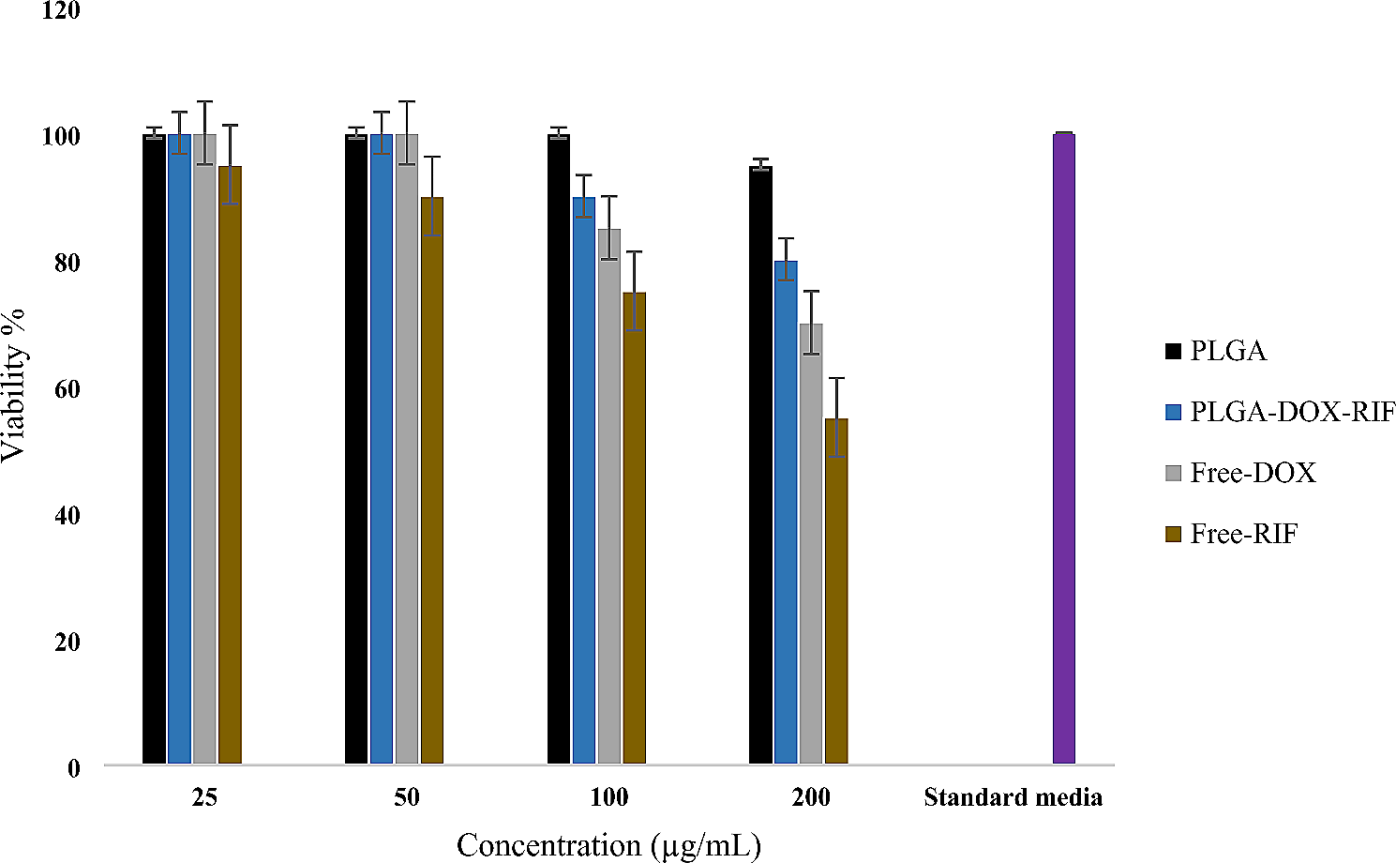
The effect of formulations on J774A.1 cells

Figure 6 illustrates the toxicity of various concentrations of Dox-Rif-PLGA@CdTe, free doxycycline, free rifampicin, and free PLGA on J774-A1 cells. The same cells were cultured in the same medium (positive controls, without any therapy). The formulations were not toxic at a concentration of 50 μg/mL. Dox-Rif-PLGA@CdTe was less toxic than free doxycycline and free rifampicin at a 200 μg/mL concentration.

### Intracellular study

**Fig. 7:**
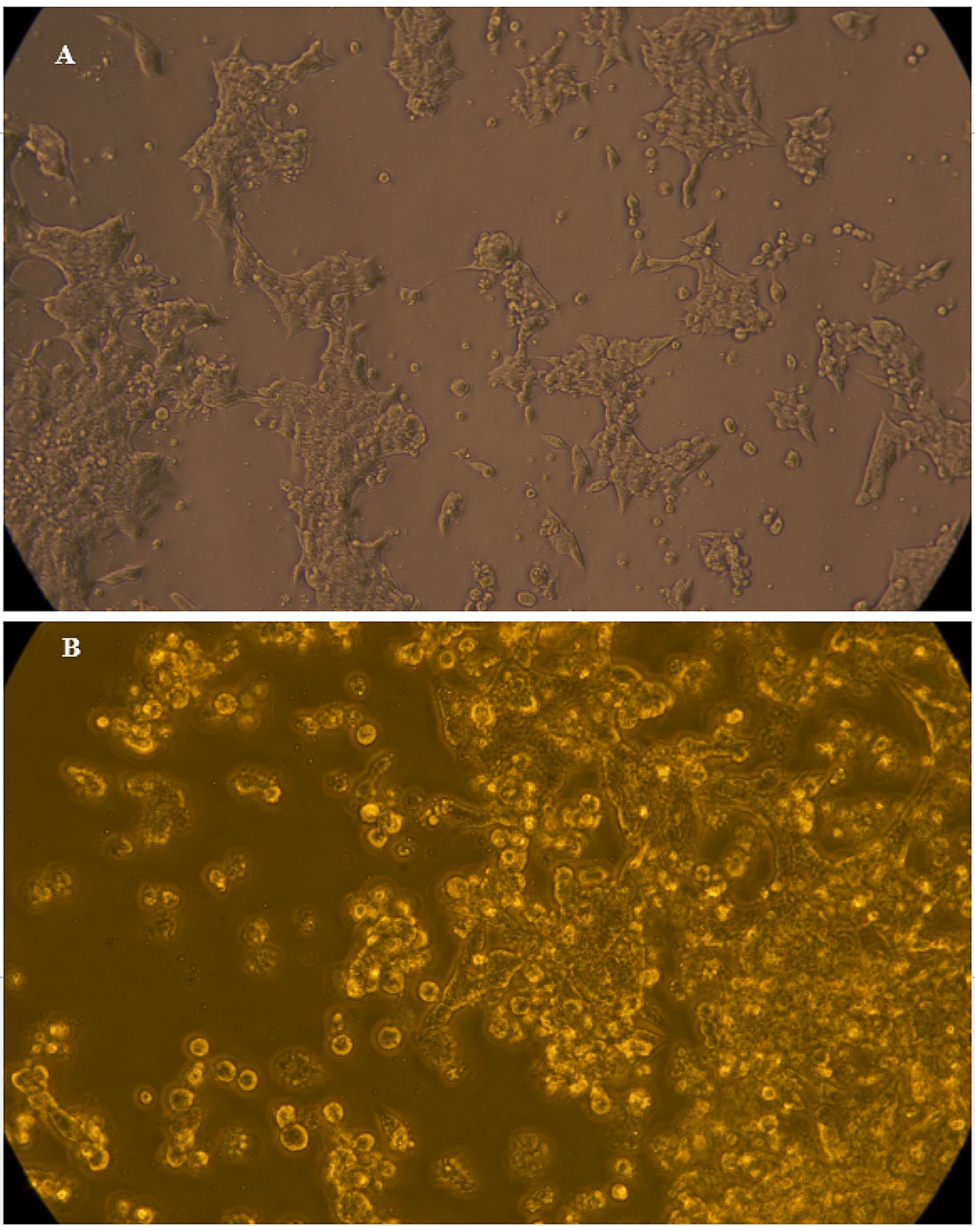
(**A**) The presence of bacteria inside macrophage cells. (**B**) Dox-Rif-PLGA@CdTe inside the macrophage cell

According to the intracellular infection investigation, Dox-Rif-PLGA@CdTe effectively decreased colony counts to as low as 3.3 logs, a significant reduction compared to free doxycycline and rifampicin treatments (*P* = 0.01). The colony counts were observed after treatment with varying concentrations of free doxycycline, free rifampicin, Dox-PLGA, Rif-PLGA, Dox-Rif-PLGA@CdTe, and free PLGA (Table 4). Figure 7 shows the presence of bacteria and labeled nanoparticles in the cell.

**Table 4:** Comparison between Free doxycycline, Free-rifampicin, and formulations against *B. melitensis* inside J774A.1 cells

Treatment	Concentration (μg/ mL)									
	200		100		50		25		12.5	
	Mean CFUs ±SEM	Log CFUs reduction	Mean CFUs ±SEM	Log CFUs reduction	Mean CFUs ±SEM	Log CFUs reduction	Mean CFUs ±SEM	Log CFUs reduction	Mean CFUs ±SEM	Log CFUs reduction
PLGA	6.4 ± 0.02	0	6.1 ± 0.04	0.3	6.3 ± 0.01	0.1	6.4 ± 0.06	0	6.2 ± 0.03	0.2
Dox-Rif-PLGA@CdTe	3.5 ± 0.04	2.9	3.4 ± 0.03	3	3.1 ± 0.04	3.3	3.2 ± 0.02	3.2	3.3 ± 0.05	3.1
Dox-PLGA	4.4 ± 0.01	2	4.2 ± 0.04	2.2	4.1 ± 0.02	2.3	3.9 ± 0.03	2.5	3.7 ± 0.04	2.7
Rif-PLGA	4.6 ± 0.04	1.8	4.3 ± 0.02	2.1	4.4 ± 0.06	2	4.1 ± 0.04	2.3	4.2 ± 0.01	2.2
Free-Dox	5.3 ± 0.05	1.1	5.2 ± 0.03	1.2	5.5 ± 0.02	0.9	5.1 ± 0.01	1.3	5.2 ± 0.02	1.2
Free-Rif	5.7 ± 0.03	0.7	5.5 ± 0.01	0.9	5.6 ± 0.03	0.8	5.3 ± 0.05	1.1	5.4 ± 0.03	1
Negative control	6.4 ± 0.02	0	6.4 ± 0.02	0	6.4 ± 0.02	0	6.4 ± 0.02	0	6.4 ± 0.02	0

## Discussion

Although antimicrobial drugs have made considerable progress in their development and efficacy, treating diseases caused by intracellular bacteria remains a formidable challenge. While drug combinations have proven effective in treating brucellosis, recurrences persist to varying degrees due to the presence of the bacterium within host cells. Consequently, developing drug delivery systems is imperative to enhance the treatment of intracellular infections [39]. This study aimed to assess the therapeutic potential of poly (lactic-co-glycolic) acid (PLGA) polymer nanoparticles loaded with doxycycline and rifampicin against *B. melitensis* in a controlled laboratory setting.

The results revealed that Dox-Rif-PLGA@CdTe significantly outperforms free doxycycline and free rifampicin in reducing bacterial counts (*p* = 0.01). Hence, the utilization of nanoparticles guarantees a continual and consistent presence of the medication at the intended target location. The synthesis technique in this study was the double emulsion evaporation method, which is a straightforward, cost-effective, and reproducible process, minimizing the use of organic solvents and surfactants [38].

Given the primary aim of transferring drugs into macrophages and cells housing the bacteria, the properties of the nanoparticles assumed a pivotal role. The diameter achieved for Dox-Rif-PLGA@CdTe (239.9 ± 25 nm) is ideal for phagocytosis by phagocytes. Singh et al. found that nanoparticle size decreased with increasing sonication time. This study further demonstrated that the size of PLGA expanded after drug loading, making Dox-Rif-PLGA@CdTe larger than free PLGA [40]. Additionally, nanoparticle size increased after lyophilization, consistent with results from Nazende Günday Türeli et al. [41]. The average Polydispersity Index (PDI) of the nanoparticles in the present study was 0.374 ± 0.015. Longer ultrasonic homogenization times led to lower PDI, producing smaller and more uniform nanoparticle sizes. However, prolonged ultrasonication was associated with decreased drug loading and encapsulation, following earlier research findings by Ito et al. Moreover, the present study found that higher surfactant concentrations yielded smaller particles, although efforts were made to limit surfactant concentration to at least 2% due to the toxic effects of high concentrations [40]. Nanoparticle synthesis characteristics can vary depending on the methods and objectives. For instance, Imbuluzqueta et al. utilized the single-emulsion solvent evaporation method to create nanoparticles and achieved a size of 299 nm [42].

In the current medical landscape, treating diseases caused by intracellular bacteria remains a formidable challenge despite significant advancements in antimicrobial drug development. While drug combinations have shown promise in brucellosis treatment, the bacterium’s intracellular presence often leads to varying degrees of relapse. Therefore, developing drug delivery systems becomes imperative to improve the management of intracellular infections. This study aimed to assess the therapeutic potential of poly (lactic-co-glycolic) acid (PLGA) polymer nanoparticles, which were conjugated with cadmium telluride quantum dots and loaded with doxycycline and rifampicin antibiotics, against *B. melitensis* in a controlled laboratory setting.

The findings indicated that Dox-Rif-PLGA@CdTe performs better than free doxycycline and rifampicin in decreasing bacterial quantities (*p* = 0.01). This implies that nanoparticles guarantee the medication’s continuous and consistent presence at the intended target location. The methodology employed in this investigation was the double emulsion evaporation technique, a straightforward, cost-effective, and reproducible procedure, thereby minimizing the utilization of organic solvents and surfactants. The properties of the nanoparticles played a pivotal role, given the primary aim of transferring drugs into macrophages and cells housing the bacteria. The size of the Dox-Rif-PLGA@CdTe nanoparticle (239.9 ± 25 nm) is ideal for phagocytosis by phagocytes. The study also showed that an increase in sonication time correlated with a reduction in nanoparticle size, aligning with findings from Singh et al. [43]. This study also demonstrated that the size of PLGA expanded after drug loading, making Dox-Rif-PLGA@CdTe larger than free PLGA. Additionally, nanoparticle size increased after lyophilization, consistent with results from Nazende Günday Türeli et al. [44]. The average Polydispersity Index (PDI) of the nanoparticles in our study was 0.374 ± 0.015. Increased ultrasonic homogenization duration significantly reduced PDI, leading to smaller and more uniform nanoparticle sizes. However, earlier research findings, such as Ito et al. [45], associated prolonged ultrasonication with decreased drug loading and encapsulation. Moreover, the present study found that higher surfactant concentrations yielded smaller particles, although efforts were made to limit surfactant concentration to at least 2% due to the toxic effects of high concentrations. The characteristics of nanoparticle synthesis can vary depending on the methods and objectives of nanoparticle production. For instance, Imbuluzqueta et al. utilized the single-emulsion solvent evaporation method to create nanoparticles and achieved a size of 299 nm.

In the study, Singh et al. (2016) in India considered their nanoparticles to be 100 nm in size due to their subcutaneous drug application [40]. Another physicochemical parameter, zeta potential, was measured at -19.2mV in this study. The negative zeta potential is crucial for enhancing particle stability as it generates repulsive forces that prevent particle aggregation over time [34].

A variety of nanoparticles are employed in the drug delivery systems. The drug loading and encapsulation levels in different nanoparticles depend on the materials used for synthesis and the preparation methods. In this study, drug loading and encapsulation efficiencies were 15.2% and 83.5% for doxycycline and 14.5% and 94.2% for rifampicin, respectively [45]. The hydrophobic nature of rifampicin was found to promote interaction with the hydrophobic part of PLGA, as observed in similar studies.

The release of drugs from nanoparticles plays a crucial role, and this study aimed to achieve a slow and controlled drug release. The drug release curves for the microspheres illustrated a relatively stable drug release rate throughout the process, without any notable burst release. The study indicated that approximately 80% of the drug was released from the synthesized nanoparticles after 100 h. While the duration of drug release from various nanoparticles varies across studies due to different methods and materials, nanoparticles generally offer extended drug release durations compared to free drugs. The findings from good diffusion and Minimum Inhibitory Concentration (MIC) tests indicate no statistically significant distinction in the efficacy of free drugs compared to the synthesized nano drug when dealing with *B. melitensis*. In these methods, free drugs exhibited superior effectiveness in the initial 24–48 h when bacteria are directly exposed to the drug. However, the nano-drug and free drug exhibited nearly equal zone diameters in good diffusion and MIC after 72 h [34].

Misra et al. [46] involved a comparison of the stability between doxycycline-loaded nanoparticles and native doxycycline over a 10-day timeframe to illustrate the diminishing efficacy of native drugs over time. The experiment was conducted on bacterial cultures, revealing that native doxycycline remained effective for only two days, whereas the nanoparticle formulation retained its efficacy for up to 10 days. This underscores the decline in the stability of native drugs over time and their diminishing antibacterial properties after two days, as evidenced by the higher bacterial colony counts [46]. Applying nanoparticles loaded with doxycycline leads to a regulated drug release, demonstrating increased efficacy against E. coli bacteria over an extended period compared to conventional doxycycline.

The assessment of toxicity holds a crucial role in the field of nano-drug synthesis and application. Considering that the ultimate objective of nanomedicines is human disease treatment, investigating their potential toxicity is paramount. The current investigation observed that Dox-Rif-PLGA@CdTe at a 50 μg/mL concentration displayed no toxicity towards J774A.1 macrophage cells, ensuring 100% cell survival. Furthermore, the toxicity of Dox-Rif-PLGA@CdTe was lower compared to free doxycycline and free rifampicin on cells at 100 and 200 μg/mL. A bacterial strain resides within a macrophage, and antibiotics do not directly contact the bacteria in the cellular study. CdTe-QDs were used to label the nanoparticles and facilitate the entry of nanoparticles into macrophages [13].

Lecaroz et al. investigated the impact of PLGA loaded with gentamicin on human monocytes, revealing better bacterial inhibition than the free drug. However, the efficacy observed was lower than that of Dox-Rif-PLGA@CdTe synthesized in the current study. In a different investigation by Seleem et al., Nanoplexes loaded with doxycycline were employed to combat *B. melitensis* within J774A.1 cells. They reported no difference between the effectiveness of the drug-loaded nanoparticles and the corresponding free drug [47].

However, this study faced several limitations. Dealing with B. melitensis presented a substantial challenge due to its pathogenic nature. Multiple trial and error iterations were essential to identify appropriate nanoparticles, resulting in an extended project duration. Moreover, the project necessitated various specialized equipment dispersed across different locations, adding complexity to the work.

### Limitations

Studying B. melitensis was very dangerous due to its pathogenic nature. Since there was a lot of trial and error to prepare a suitable nanoparticle, the project’s duration was long, and the high price of PLGA and the low loading rate of this nanoparticle were the other limitations.

### Future perspectives

1. Investigating the antimicrobial effect of Dox-Rif-PLGA@CdTe in *invivo* conditions.
2. Evaluating the effect of Dox-Rif-PLGA@CdTe on different tissues of the body, especially the kidney and liver.
3. Examining the effect of Dox-Rif-PLGA@CdTe on other bacterial species such as *Brucella abortus.*
4. Assessing the plasma concentration of rifampicin and doxycycline.

## Conclusion

The double emulsion method was employed in the formulation of Dox-Rif-PLGA@CdTe, resulting in nano drug carriers of an optimal size that macrophages can readily engulf. Consequently, these nano drug carriers exhibit a higher capability to hinder intracellular B. melitensis infection than free drugs. Overall, the findings underscore the potential of nanoplatforms in enhancing the effectiveness of conventional anti-brucellosis medications. Hence, utilizing Nano drug carriers should be considered a viable option for brucellosis treatment. Nevertheless, it is imperative to conduct further research to thoroughly examine the interactions between nano drug carriers and eukaryotic cells.

### Electronic supplementary material

Below is the link to the electronic supplementary material.

Supplementary Material 1

## Data Availability

Data is provided within the manuscript or supplementary information files.
