# Supplementary material for: Co-delivery of doxycycline and rifampicin using CdTe-labeled poly (lactic-co-glycolic) acid for treatment of Brucella melitensis infection
Source: BMC Chem. 2024 May 15;18(1):100. doi: 10.1186/s13065-024-01200-8 (PMC11097527; doi:10.1186/s13065-024-01200-8)
Supplement: Supplementary file 1 — Supplementary Material 1 [file 13065_2024_1200_MOESM1_ESM.docx]

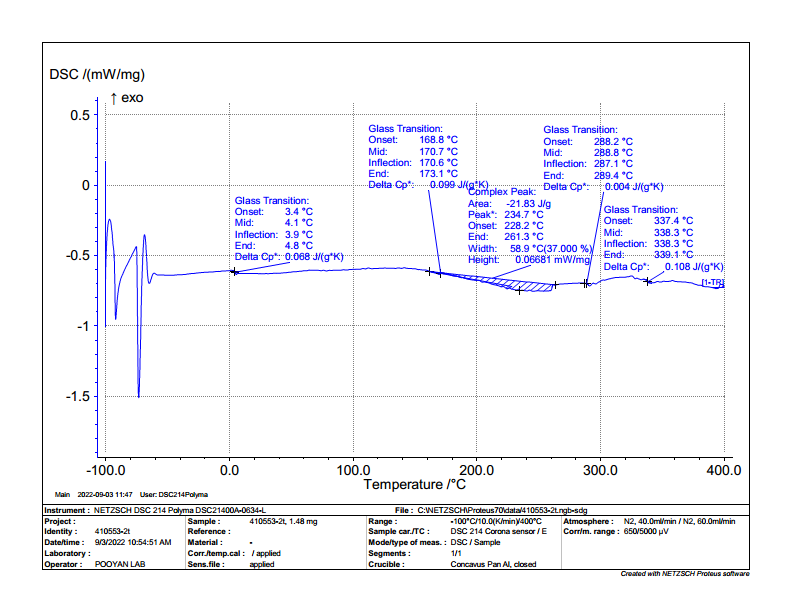


Supplementary figures 1: DSC thermogram of Physical mix 1


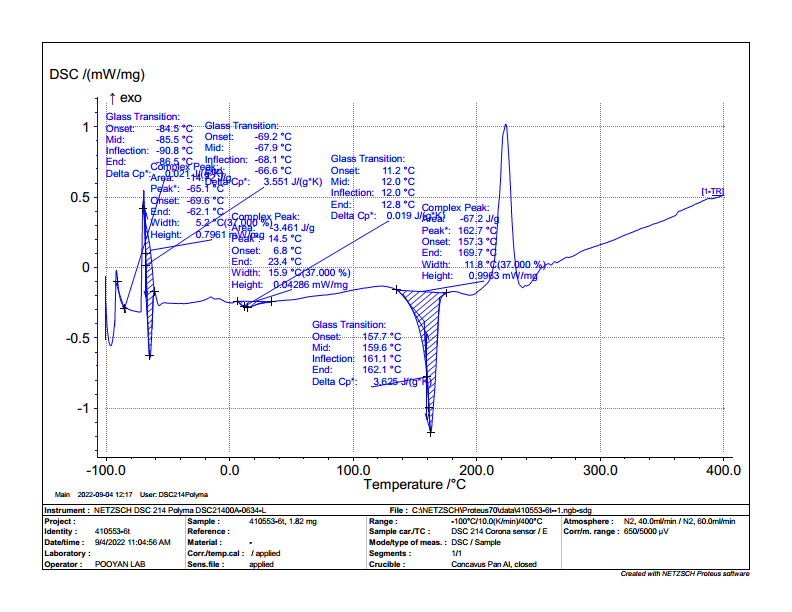


Supplementary figures 2: DSC thermogram of free-DOX 1


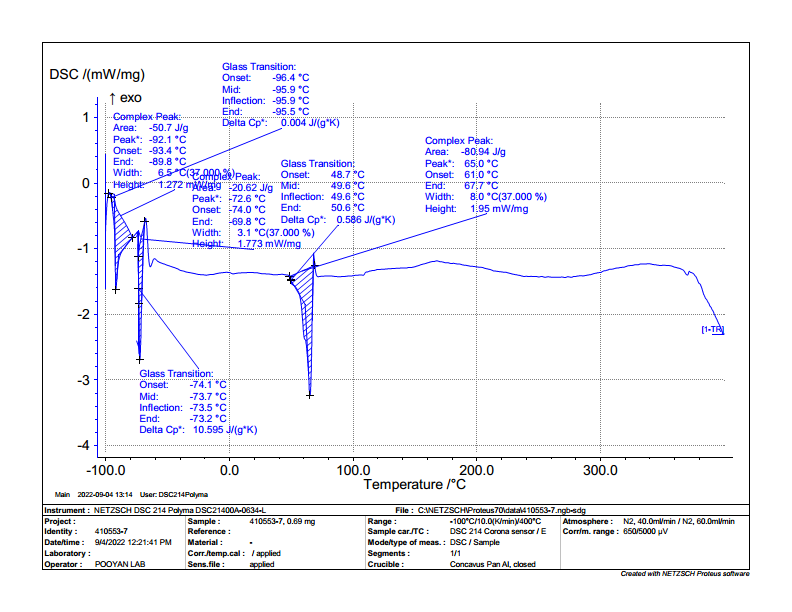


Supplementary figures 3: DSC thermogram of free-PLGA


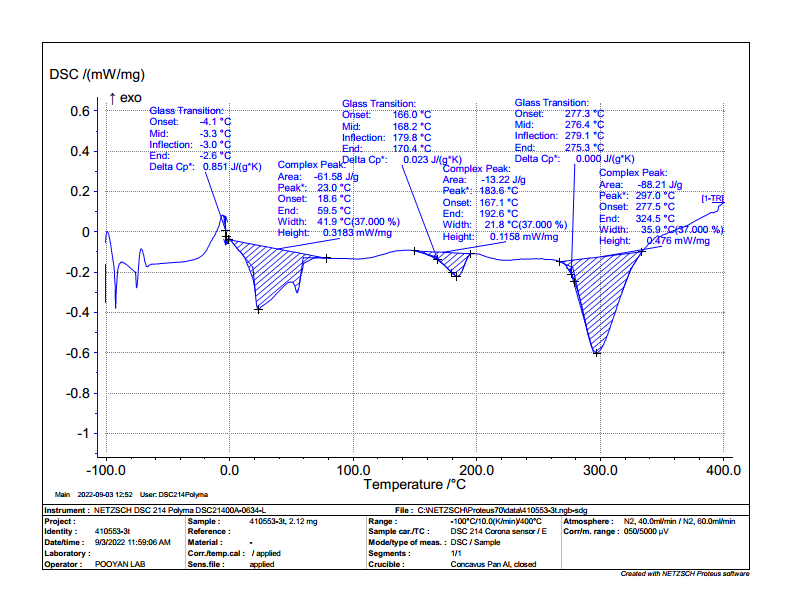


Supplementary figures 4: DSC thermogram of free-RIF


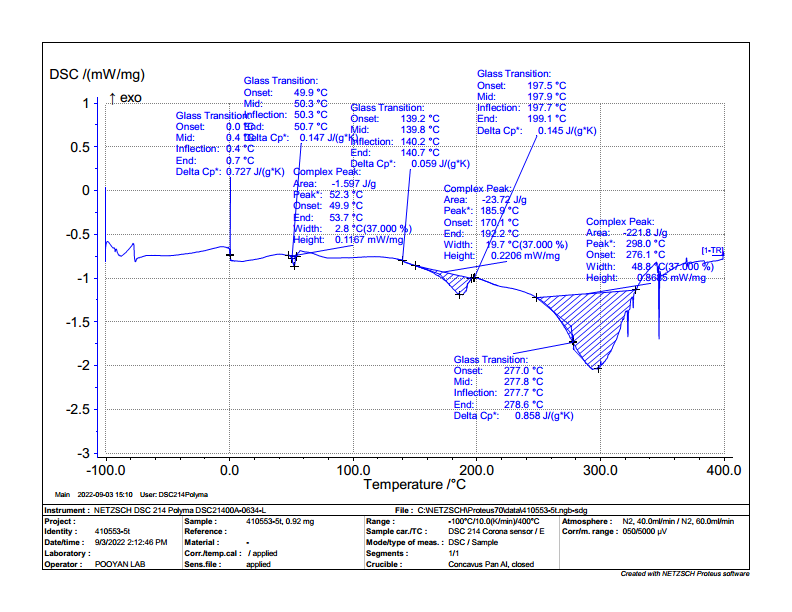


Supplementary figures 5: DSC thermogram of PLGA-DOX


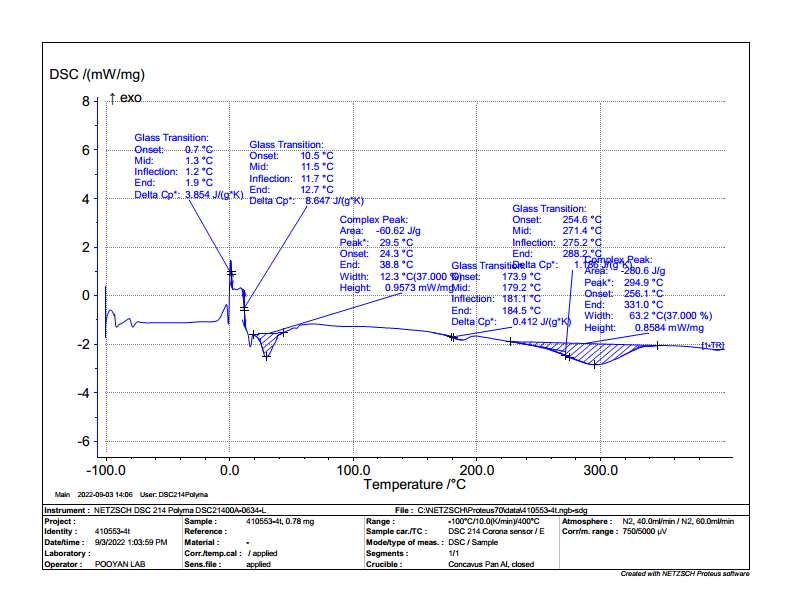


Supplementary figures 6: DSC thermogram of PLGA-DOX-RIF
